# miR-93-3p inhibition suppresses clear cell renal cell carcinoma proliferation, metastasis and invasion

**DOI:** 10.18632/oncotarget.20458

**Published:** 2017-08-24

**Authors:** Lu Wang, Guang Yang, Xiangwei Zhu, Ziqi Wang, Hongzhi Wang, Yang Bai, Pengcheng Sun, Li Peng, Wei Wei, Guang Chen, Guangbin Li, Andrey A. Zamyatnin, Peter V. Glybochko, Wanhai Xu

**Affiliations:** ^1^ Department of Urology, The Fourth Hospital of Harbin Medical University, Harbin, Heilongjiang Province, P.R. China; ^2^ Department of Neurosurgery, The First Affiliated Hospital of Harbin Medical University, Harbin, Heilongjiang Province, P.R. China; ^3^ Institute of Molecular Medicine, Sechenov First Moscow State Medical University, Moscow, Russia; ^4^ A.N. Belozersky Institute of Physico-Chemical Biology, Lomonosov Moscow State University, Moscow, Russia

**Keywords:** ccRCC, miR-93-3p, PEDF, apoptosis, migration

## Abstract

miRNA dysregulation is associated with many human diseases, including cancer. This study explored the effects of miR-93-3p on clear cell renal cell carcinoma (ccRCC). We found that miR-93-3p is upregulated an average of 38-fold in 138 ccRCC specimens compared to matched normal kidney tissues, which correlated with poor patient outcome. miR-93-3p inhibition reduced ccRCC cell growth, invasion, and migration *in vitro* and in a mouse xenograft model. A search of the TargetScan, miRanda, and PicTar databases revealed that miR-93-3p is predicted to regulate pigment epithelium-derived factor (PEDF). A direct PEDF-miR-93-3p interaction was confirmed via dual-luciferase reporter assays. Like miR-93-3p inhibition, PEDF overexpression induced cell apoptosis and inhibited migration and invasion. Additionally, co-transfection with PEDF siRNA reversed the effects of miR-93-3p inhibition in ccRCC cells. Thus, miR-93-3p is a likely ccRCC oncogene that acts by regulating PEDF. These results suggest that miR-93-3p may predict ccRCC patient clinical outcome and serve as a novel anti-ccRCC therapeutic target.

## INTRODUCTION

Renal cell carcinoma is one of the 10 most common cancers in both men and women, and clear cell renal cell carcinoma (ccRCC) is the most frequent histological subtype. ccRCC represents approximately 70% of renal cell carcinomas. Even with surgical resection, 20–50% of patients diagnosed with localized tumors develop local recurrence or metastases within three years, and five-year survival in patients with stage IV disease is approximately 10% [1–4]. Current first line treatments for metastatic ccRCC are limited to the angiogenesis and tyrosine kinase inhibitors, pazopanib and sunitinib. Anti-angiogenic therapy is efficacious against metastatic ccRCC. However, the ability of anti-angiogenic drugs to delay tumor progression and extend patient survival is limited, due to either innate or acquired drug resistance [5–7]. Despite the application of multimodal therapies, including surgical excision and chemotherapy, ccRCC patient prognoses remain unfavorable. Thus, novel predictive markers and effective therapeutic strategies against this malignancy are urgently needed.

MicroRNAs (miRNAs) are a class of endogenous, small noncoding RNAs that regulate gene expression by directly degrading or inhibiting translation of target mRNAs through base pairing to partially complementary sites. Dysregulation of miRNA expression and activity is associated with a variety of human diseases, and many miRNAs are potential ccRCC prognostic markers and therapeutic targets [3].

Pigment epithelium derived factor (PEDF) is a multifunctional serpin with antiangiogenic activity in tumor cells [8, 9]. This study showed for the first time that miR-93-3p inhibitor induced ccRCC cell apoptosis, inhibited metastasis, and upregulated PEDF expression. We also found that miR-93-3p overexpression was associated with poor prognosis in a large ccRCC patient cohort from a single institution.

## RESULTS

### miR-93-3p expression correlates with ccRCC patient outcome

**Figure 1:**
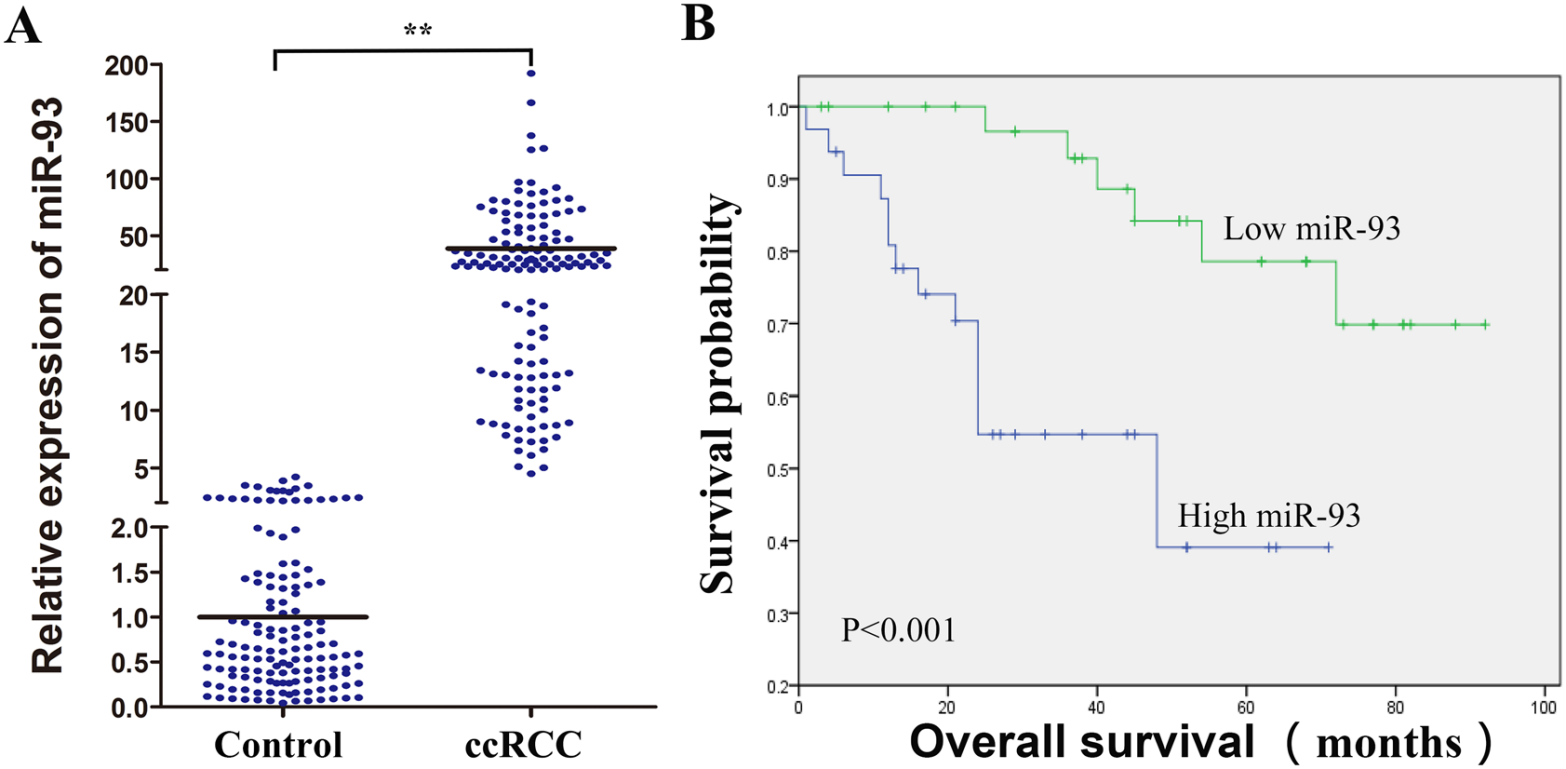
Clinical significance of miR-93-3p in ccRCC patients miR-93-3p expression was calculated using the comparative Ct method. Relative expression intensity values were calculated as 2^-∆∆^Ct. miR-93-3p expression in 138 FFPE normal and ccRCC specimens **(A)**. Control refers to normal kidney tissues, and miR-93-3p levels were normalized to controls. Correlation of miR-93-3p expression with ccRCC patient overall survival **(B)**. “NC”, “miR” and “anti-miR” represented negative control, miR-93-3p and miR-93-3p inhibitor respectively. ^**^P<0.001.

Many microRNAs regulate ccRCC cell functions [10, 11]. We detected miR-93-3p expression (normalized to matched normal kidney tissues) in 138 formalin-fixed, paraffin-embedded (FFPE) ccRCC specimens via qRT-PCR. miR-93-3p was upregulated 38-fold on average in ccRCC specimens compared to normal kidney tissues (Figure 1A). miR-93-3p expression was correlated with patient survival, and miR-93-3p upregulation was associated with poor prognosis (Figure 1B). miR-93-3p relationships with clinical variables (sex, age, tumor size, location, and Fuhrman grade and pT status) were assessed using the Cox proportional hazard regression model. Average patient age (57.9 years old ≈ 60), 4cm tumor size (the Urological Diseases Guide indicates that tumor size at T1a stage is ≤4cm) and the average levels of miR-93-3p were the clinical variables in our ccRCC patients. Univariate and multivariate analyses showed that miR-93-3p level was an independent predictor of ccRCC patient overall survival (Table 1).

**Table 1:** Univariate and multivariate Cox regression analysis of overall survival in archival ccRCC patients

Variables	No. of patients (%)		Univariate analysis			Multivariate analysis		
			HR	95% CI	P value	HR	95% CI	P value
**Sex**					0.508			0.889
Female	28(42.42%)	**Male vs female**	0.749	0.317-1.765		1.069	0.419-2.725	
Male	38(57.58%)							
**Age (y)**					0.936			0.515
≤60	38(57.58%)	**>60 vs ≤60**	1.036	0.439-2.442		1.356	0.541-3.399	
>60	28(42.42%)							
**Tumor size** (cm)					0.834			0.470
≤4	20(30.30%)	**>4 vs ≤4**	0.960	0.655-1.407		1.205	0.726-2.002	
>4	32(48.48%)							
**Location**					0.385			0.267
Left	30(45.45%)	**Right vs Left**	0.684	0.290-1.611		0.601	0.245-1.476	
Right	36(54.55%)							
**Fuhrman grade**					0.237			0.075
I, II	45(68.18%)	**III, IV vs I, II**	1.462	0.779-2.747		1.995	0.933-4.264	
III, IV	18(27.27%)							
**pT status**					0.002			0.026
pT1/T2	51(77.30%)	**pT1/T2 vs pT3/T4**	4.509	1.751-11.611		3.524	1.164-10.674	
pT3/T4	15(22.70%)							
**miR-93 expression**					0.002			0.008
Low	34(51.52%)	**High vs Low**	5.281	1.886-14.786		4.619	1.488-14.338	
High	32(48.48%)							

HR, hazard ratio.

### miR-93-3p regulates ccRCC cell proliferation

**Figure 2:**
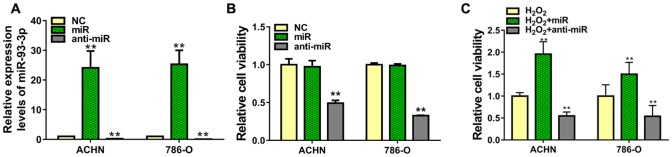
Effect of miR-93-3p on cell viability *in vitro* miR-93-3p expression was quantified using qRT-PCR **(A)** and cell viability was determined via MTT assay **(B** and **C)** in ACHN and 786-O cells 48 h after transfection with miR-93-3p mimics, anti-miR-93-3p, or NC oligo. ^**^P<0.001.

To explore the role of miR-93-3p in tumorigenesis, we transfected ACHN or 786-O cells with negative control (NC), miR-93-3p, or anti-miR-93-3p. Transfection success was assessed via qRT-PCR after 48 h, and data were normalized to NC-transfected cells. Mimic-transfected cells had >20-fold increased miR-93-3p levels, while anti-miR-93-3p showed decreased miR-93-3p levels compared with the NC (Figure 2A). Cell viability was analyzed by MTT assay 48 h post-transfection. Compared to the NC, miR-93-3p overexpression did not impact cell viability, whereas anti-miR-93-3p transfection decreased ACHN and 786-O cell proliferation (Figure 2B). We then assessed the miR-93-3p protective function in ACHN and 786-O cells in an injury environment. ccRCC cells transfected with NC, miR-93-3p, or anti-miR-93-3p were treated with H_2_O_2_. miR-93-3p transfection increased treated cell viability 1.5-2-fold over controls (H_2_O_2_ + NC), while anti-miR-93-3p again inhibited viability (Figure 2C).

### Anti-miR-93-3p promotes cell apoptosis and suppresses invasion and metastasis *in vitro*

**Figure 3:**
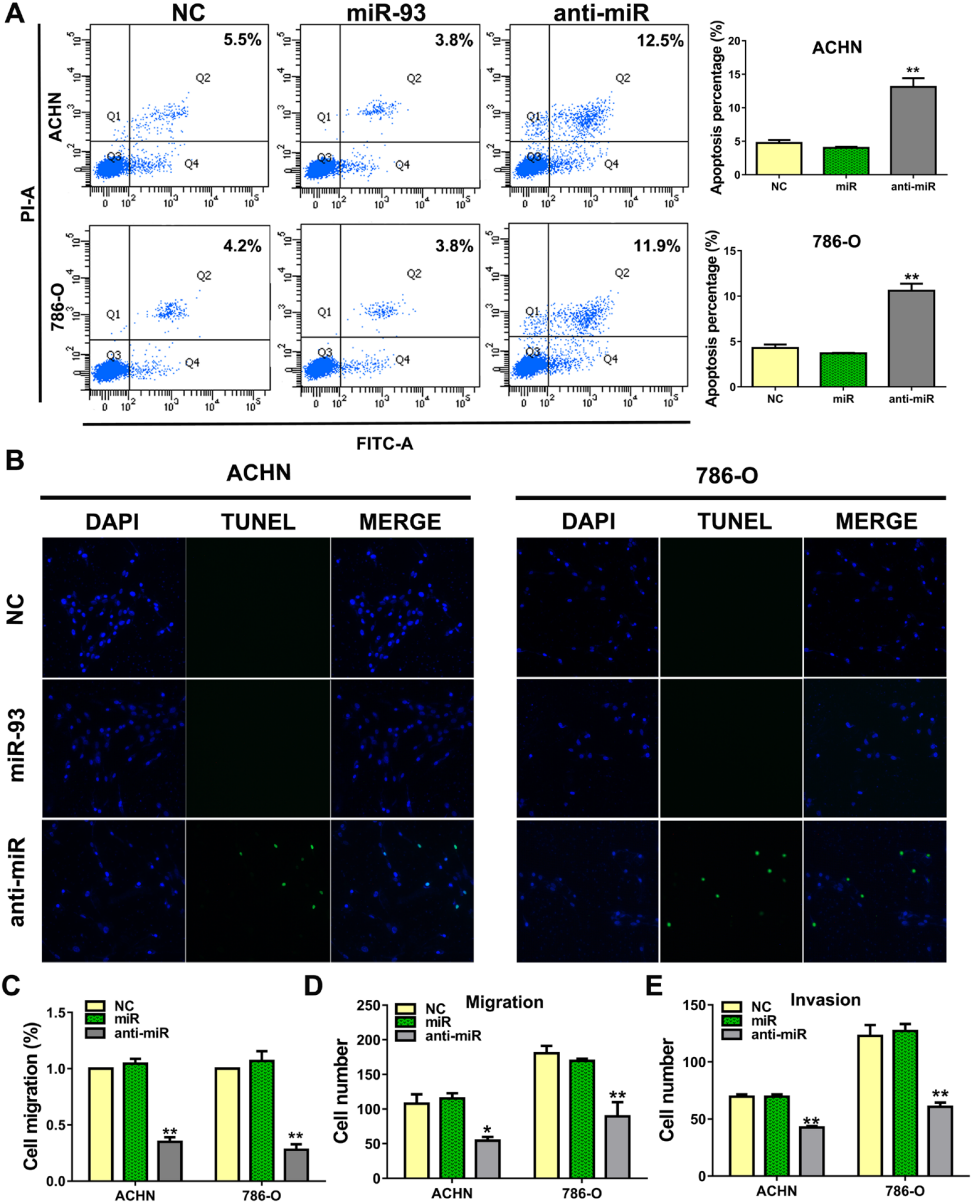
miR-93-3p suppresses ccRCC cell apoptosis and promotes migration ACHN and 786-O cells were stained with Annexin V and PI, and apoptosis was analyzed via flow cytometry **(A)**. 10^4^ cells were detected per sample. Apoptosis was detected by TUNEL staining after transfection for 48 h **(B)**. Apoptotic cells are green, and nuclei are stained blue with DAPI. Wound healing assay showing transfected cell migration **(C)**. Wound widths were normalized to NC-transfected cells. Wound healing percentage was calculated as: (wound width at 0 h - wound width at 48 h) / wound width at 0 h. ACHN and 786-O cell transwell migration **(D)** and invasion assays **(E)**. ^*^P<0.05, ^**^P<0.001.

To determine the effect of anti-miR-93-3p on apoptosis, we stained ACHN and 786-O cells with Annexin V and propidium iodide (PI). anti-miR-93-3p-transfected cells exhibited more Annexin V positivity (apoptosis) than NC-transfected cells. Flow cytometry results showed that 13.1±2.3% and 10.6±1.4% of anti-miR-93-3p-transfected ACHN and 786-O cells, respectively, were apoptotic (Figure 3A). TUNEL assay results also showed greater levels of apoptosis in anti-miR-93-3p-transfected cells than in NC-transfected cells (Figure 3B).

We then performed a wound-healing assay to assess the impact of anti-miR-93-3p on ACHN and 786-O cell migration. anti-miR-93-3p inhibited cell migration into the wounded area (Figure 3C). In transwell migration assays, 107.6±27.1 ACHN cells and 180.5±21.8 786-O cells traversed the membrane under control conditions. anti-miR-93-3p transfection reduced these numbers to 54.3±11.2 and 89.5±41.3 in ACHN and 786-O cells, respectively (Figure 3D). Cell invasion was assessed using a three-dimensional matrigel-coated filter. anti-miR-93-3p transfection decreased the number of invaded ACHN and 786-O cells compared with the NC. While 69.5±4.0 ACHN cells and 122.8±19.3 786-O cells traversed the matrigel-coated membrane under control conditions, anti-miR-93-3p transfection decreased these numbers to 42.5±2.5 and 58.0±6.4 cells, respectively (Figure 3E). These results demonstrated that anti-miR-93-3p increased ACHN and 786-O cell apoptosis and decreased migration and invasion.

### miR-93-3p directly targets PEDF

**Figure 4:**
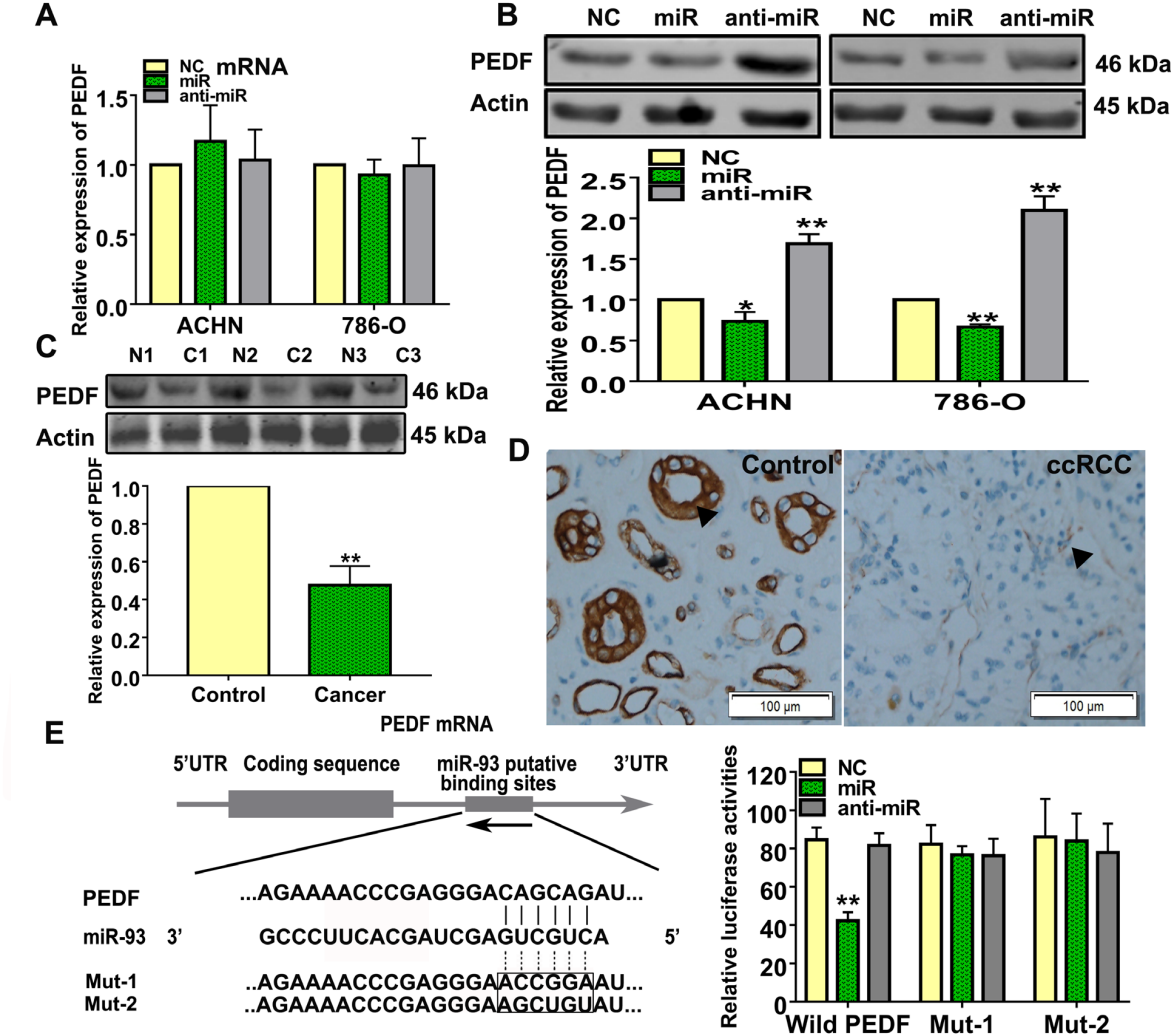
miR-93-3p directly targets PEDF PEDF expression was measured using qRT-PCR **(A)** and western blotting **(B)** after NC, miR-93-3p, or anti-miR-93-3p transfection for 48 h in ACHN and 786-O cells. Actin was used as a loading control in western blots. Experiments were repeated at least three times with duplicate samples. PEDF levels were measured in ccRCC patient samples via western blotting **(C)**. Photomicrographs showing representative hematoxylin and eosin staining and PEDF immunohistochemical analysis results in human ccRCC and normal kidney tissues **(D)**. The potential interaction between miR-93-3p and putative binding sites in the *PEDF* 3’-UTR. NC, miR-93-3p, or anti-miR-93-3p were co-transfected with the wild type PEDF 3’-UTR, Mut1, or Mut2 into HEK293 cells for 48 h. Luciferase activities were analyzed relative to the NC-transfected group **(E)**. Data were presented as means ± SEM. Experiments (C-E) were repeated six times each with duplicate samples. ^*^P<0.05, ^**^P<0.001.

We searched the supplementary databases, TargetScan, miRanda, and PicTar, for potential miR-93-3p targets, and found that miR-93-3p was predicted to regulate *PEDF*. Neither miR-93-3p nor anti-miR-93-3p transfection affected PEDF mRNA levels in ACHN and 786-O cells (Figure 4A). However, PEDF proteins were affected by miR-93-3p and anti-miR-93-3p transfections in both ACHN and 786-O cells (Figure 4B). Western blot analysis also revealed that PEDF was downregulated in ccRCC patient tumor tissues compared to normal kidney tissues (Figure 4C). Immunohistochemistry results indicated that ccRCC specimens exhibited a weakly brown staining for PEDF (Figure 4D).

To determine whether there was a direct interaction between miR-93-3p and PEDF, the wild type or mutant *PEDF* 3’-UTR was inserted into the dual-luciferase reporter plasmid. NC, miR-93-3p mimics, or anti-miR-93-3p were then co-transfected with the constructed plasmid into HEK293 cells. miR-93-3p inhibited relative luciferase activity in the reporter plasmid containing the wild type, but not mutants, *PEDF* 3’-UTR, demonstrating that miR-93-3p directly targets PEDF (Figure 4E).

### PEDF overexpression reversed the effects of miR-93-3p inhibition in ccRCC cells

**Figure 5:**
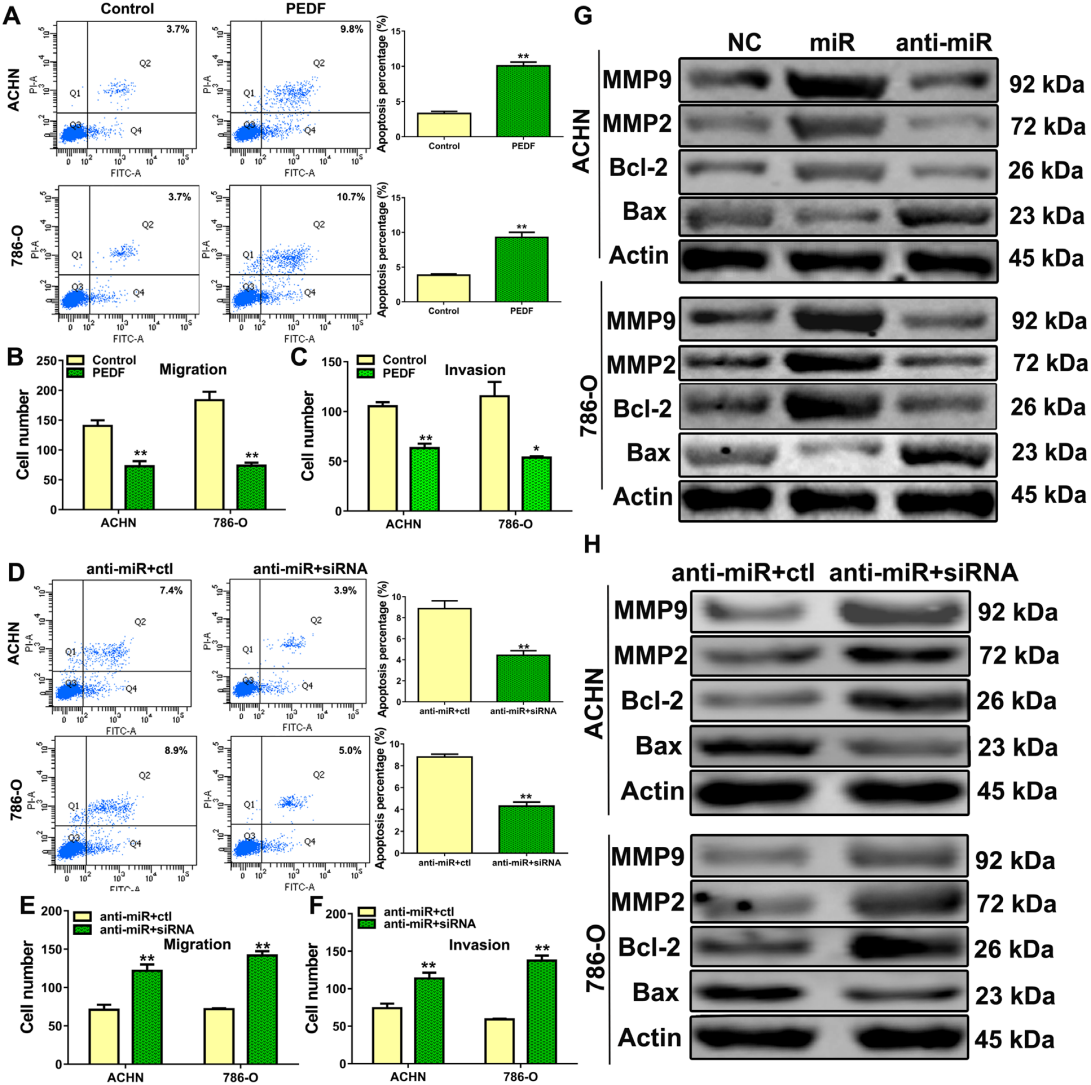
The role of PEDF in ccRCC cells ACHN and 786-O cells were transfected with control or PEDF vector and stained with Annexin V and PI, and apoptosis was analyzed via flow cytometry **(A)**, transwell migration **(B)** and invasion assays **(C)** were performed in PEDF-transfected cells. Cell apoptosis was analyzed via flow cytometry after PEDF siRNA was co-transfected with anti-miR-93-3p into ACHN and 786-O cells **(D)**. Co-transfected ACHN and 786-O cell transwell migration **(E)** and invasion assays **(F)**. Apoptosis-related proteins **(G)** and MMP2/9 **(H)** were analyzed. All experiments were repeated at least three times with duplicate samples.^*^P<0.05, ^**^P<0.001.

To determine whether PEDF mediated miR-93-3p or anti-miR-93-3p activities, ACHN and 786-O cells were transfected with a control or PEDF expression plasmid. Similar to the effects of anti-miR-93-3p, PEDF overexpression induced cell apoptosis (Figure 5A) and inhibited migration and invasion (Figure 5B–5C). Additionally, co-transfection with PEDF siRNA reversed the effects of anti-miR-93-3p transfection in ACHN and 786-O cells (Figure 5D–5F).

Anti-miR-93-3p also affected expression of several apoptosis-related proteins and matrix metalloproteinases (MMPs). anti-miR-93-3p transfection increased Bax, and decreased Bcl-2, MMP2, and MMP9 levels in ACHN and 786-O cells compared to controls (Figure 5G). These proteins were also detected after PEDF siRNA co-transfection with anti-miR-93-3p into ACHN and 786-O cells. PEDF siRNA co-transfection abrogated the anti-miR-93-3p effects in these cells compared to controls (Figure 5H).

### Anti-miR-93-3p suppressed ccRCC cell tumorigenesis and metastasis *in vivo*

**Figure 6:**
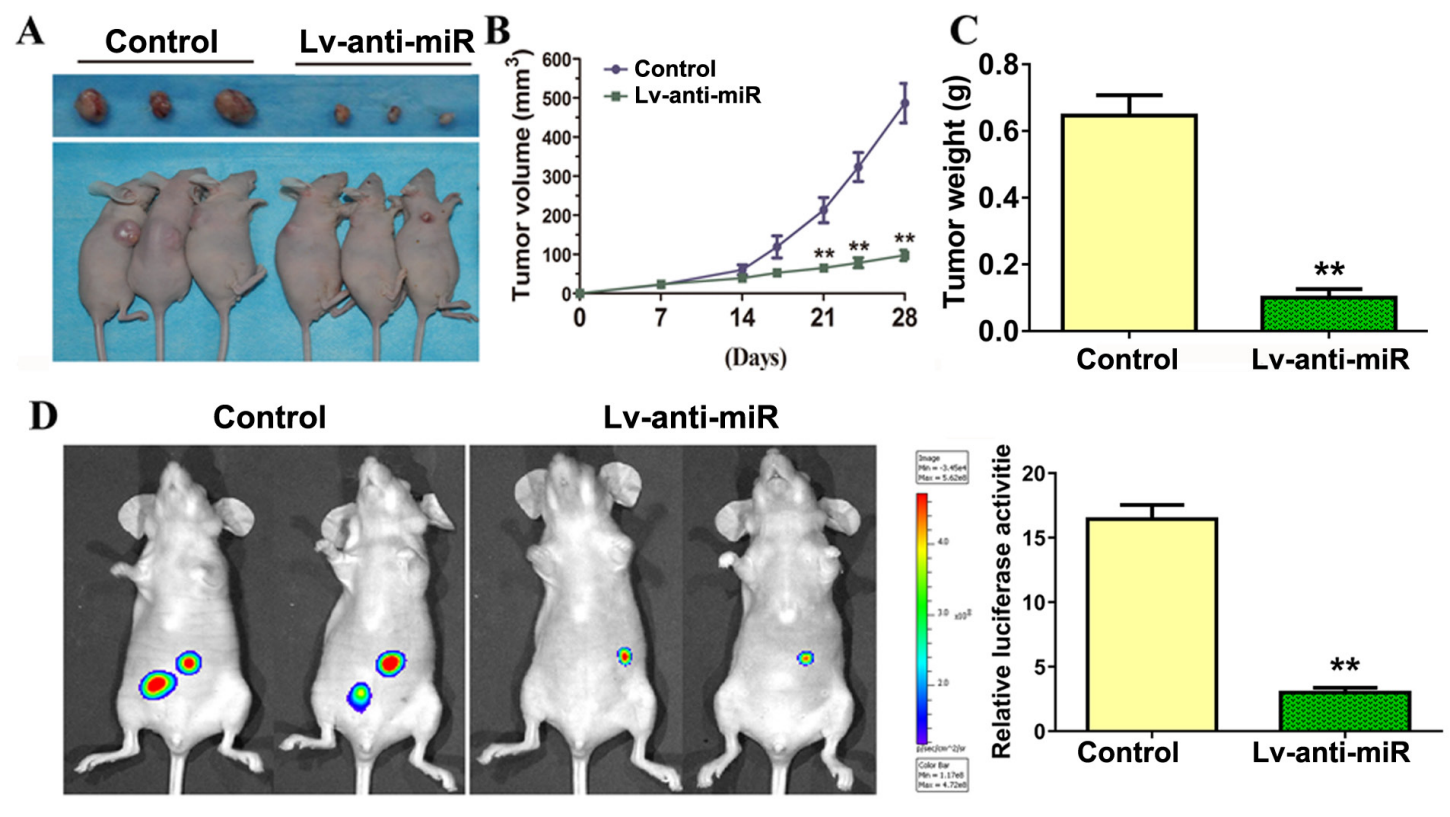
miR-93-3p inhibition suppresses ccRCC tumorigenesis *in vivo* Representative tumors excised from mice in each group implanted with 786-O cells for four weeks **(A)**. Growth inhibition of human tumor xenografts in nude mice **(B)**. Excised tumor weight on day 28 **(C)**. Four weeks following injection of tumor cells, mice were injected intraperitoneally with luciferin and imaged using a Xenogen IVIS imaging system and bar graph shows luciferase activity for both groups of mice using data from all mice tested **(D)**. n=6, ^**^P<0.001.

786-O cells were implanted subcutaneously in the flanks of nude mice, which were then injected with either control or anti-miR-93-3p lentivirus. anti-miR-93-3p injection inhibited tumor growth and reduced tumor weights and sizes compared to the control (Figure 6A–6C).

786-O cells transduced to express luciferase were injected into mouse tail veins, followed by control or anti-miR-93-3p lentivirus. Four weeks following tumor cell injection, mice were injected intraperitoneally with luciferin and imaged using a Xenogen IVIS imaging system to detect tumor cell metastases. Multiple tumors were present in all mice. anti-miR-93-3p suppressed 786-O cell metastasis and proliferation (Figure 6D). These data indicated that miR-93-3p knockdown may serve as a potential anti-ccRCC therapeutic strategy.

## DISCUSSION

This study presents the first evidence that miR-93-3p is overexpressed in ccRCC. miR-93-3p upregulation was correlated with poor prognosis and shorter overall survival time in a large ccRCC patient cohort. We found that miR-93-3p inhibition induced ccRCC cell apoptosis and decreased invasion and metastasis *in vivo* and *in vitro*. We also showed that miR-93-3p inhibits PEDF expression by binding the *PEDF* 3’-UTR, and miR-93-3p and PEDF levels are negatively correlated in ccRCC patients. The effects of miR-93-3p knockdown in ccRCC cells were abrogated by PEDF siRNA. Thus, the oncogenic activities of miR-93-3p in ccRCC are likely mediated by PEDF. Additionally, ccRCC tumor imaging in live mice demonstrated that miR-93-3p inhibition suppressed tumor growth and metastasis *in vivo*.

Many ectopically expressed miRNAs have been shown to promote carcinogenesis by regulating tumor cell proliferation, apoptosis, and metastasis. Inhibition of these miRNAs represents a new avenue for anti-cancer treatments [12–14]. Thus far, only two published studies directly reported on miR-93 in ccRCC, and both focused on miR-93-5p. Shi, *et al.* found that TGF-β induces RBL2 expression and arrests renal cancer cell growth by downregulating miR-93-5p, and indirectly showed that miR-93-5p suppression induces G1 phase arrest. Wulfken, *et al.* demonstrated that miR-93-5p is upregulated in renal cancer patient serum and tissues, but the miR-93-5p function was not well understood [15, 16].

miRNAs also modulate renal carcinoma tumorigenesis and their expression patterns can distinguish between renal carcinoma subtypes [10, 17, 18]. While miR-93 is upregulated in several solid tumors and is positively correlated with malignancy [19–21], its oncogenic mechanisms in ccRCC have not been addressed. The present study associated miR-93-3p with ccRCC patient survival via univariate and multivariate analyses, and assessed the biological functions of miR-93-3p *in vitro*. ccRCC cell transfection with an miR-93-3p expression vector did not affect cell viability or apoptosis, possibly due to the already-high viability and low apoptosis levels of these cells, but did decrease damage induced by H_2_O_2_ treatment. Additionally, miR-93-3p inhibition suppressed ccRCC cell motility and invasion, and increased apoptosis. Our results demonstrate that miR-93-3p is an oncogene in ccRCC, and may act by regulating PEDF.

PEDF is a serpin with multiple biological functions, including anti-angiogenic, tumor growth inhibitory, cell apoptosis promoting, and anti-metastatic activities [9]. PEDF inhibits melanoma cell growth by inducing apoptotic cell death, and PEDF loss in melanoma cells is associated with metastasis and poor prognosis [22]. PEDF inhibits breast cancer growth and metastasis by downregulating fibronectin via laminin receptor/AKT/ERK signaling [23]. PEDF also triggers lung cancer cell apoptosis by regulating p53 protein-driven Fas ligand and Fas protein cell surface translocation [24]. Mirochnik, *et al.* found that a short PEDF peptide effectively blocked renal carcinoma cell growth *in vivo* [25]. Jiang, *et al.* reported that most ccRCC patients are positive for PEDF, which is an independent favorable prognostic factor inversely correlated with grade and pT stage [26].

In conclusion, we presented the first evidence that miR-93-3p inhibition suppresses ccRCC cell proliferation, metastasis, and invasion, and promotes apoptosis by regulating PEDF. We confirmed that miR-93-3p overexpression is common in ccRCC. Our results suggest that miR-93-3p may predict ccRCC patient clinical outcome and serve as a novel anti-ccRCC therapeutic target.

## MATERIALS AND METHODS

### Patients with ccRCC and their surgically excised specimens

Surgically excised normal and tumor specimens were collected from 138 clear cell renal cell carcinoma patients (matched normal kidney tissues) who underwent surgery in the Department of urinary surgery at the Forth Affiliated Hospital of Harbin Medical University in China between 2008 and 2014. Tissue samples were routinely processed for histological diagnosis in strict accordance with World Health Organization criteria. Normal kidney tissues were obtained from normal adjacent tissues away from tumor tissues or non-neoplastic kidney diseases and were histologically confirmed to be free of any pathological lesions. The follow-up data were available for 66 cases. The study was approved by the Institutional Review Board of Harbin Medical University, and the participants gave informed consent.

### Cell culture

The human renal clear cell carcinoma (ccRCC) 786-O, ACHN cells and human embryonic kidney (HEK) 293 cells were obtained from the American Type Culture Collection (ATCC). 786-O cell is maintained in RPMI-1640 Medium. ACHN and HEK293 are maintained in DMEM Medium. The medium supplemented with 10% fetal bovine serum (Invitrogen, USA).

### MicroRNA, siRNA and plasmid transfection

miR-93-3p mimic, negative control miRNA (NC), miR-93-3p antisense inhibitor (anti-miR-93-3p) and PEDF siRNA were synthesized by Shanghai GenePharma Company. PEDF overexpression pcDNA3.1 vector was synthesized by Company (Genechem, China). ccRCC cells were transfected with miR-93-3p, NC, anti-miR-93-3p, siRNA or plasmid using Lipofectamine 2000 reagent (Invitrogen, USA) following the manufacturer’s instructions.

### Cell viability assays

The cells were cultured in 96-well plates, and each well was seeded with 4×10^3^ cells. After transfection with 50 nM miR-93-3p, NC, anti-miR-93-3p, the viability of the cancer cells was detected with 3-(4, 5-dimethylthiazol-2-yl)-2, 5-diphenyl tetrazolium bromide (MTT; Roche; Switzerland). 20 μl of MTT solution (5 mg/ml, Sigma, USA) was added to each well, and the mixtures were incubated for 4 h at 37°C. Then, 200 μl of dimethyl sulfoxide (DMSO) was added to the wells. The absorbance was measured using an ELISA plate reader at 490 nm. A high absorbance indicated a greater viability.

### Apoptosis assay

The apoptosis ratio was analyzed using flow cytometry and TUNEL.

The cells were transfected for 48 h then collected and washed twice with cold PBS (phosphate buffered saline). The cells were resuspended in 500 μl of binding buffer at a concentration of 10^6^ cells/ml and then mixed with 10 μl of Annexin V (BestBio, China) for 15 min in the dark at room temperature (RT). Then 5 μl of PI was added to the cells. After incubation for 5 min in the dark at RT, the samples were analyzed using a FACSAria flow cytometry (BD Biosciences, USA).

The TUNEL assay was performed according to the protocol of death detection kit (Roche, Switzerland). Briefly, the cells were fixed in 4% paraformaldehyde in PBS, washed three times with PBS, and washed with 3% H_2_O_2_ in methanol for 10 min at RT. After incubating with 0.1% Triton X-100 in 0.1% sodium citrate for 2 min on ice, the cells were incubated with the TUNEL reaction mixture for 1 h and then with DAPI for 5 min in the dark at RT. Fluorescence microscope (Nikon, Japan) were used for data analysis.

### Wound healing assay

Cells were seeded in six-well plates in culture medium. Cells were grown to 70% confluence, rinsed with phosphate-buffered saline (PBS), and then starved for 12 hours in serum-free medium. A sterile 200 μL pipette tip was used to create wounds. Furthermore, cells were transfected with NC, miR-93-3p and anti-miR-93-3p respectively. The migration of the cells across the wound line was assessed after 48 hours.

### Migration and invasion assay

Cellular invasiveness was quantified using a modified Matrigel Boyden chamber assay, as previous described [27]. 1×10^5^ cells seeded into upper chambers. The chambers were then inserted into transwell apparatus (Costar, USA). The upper chambers were coated with Matrigel (BD Biosciences, USA) when cell invasion assay was done. Medium with 10% FBS was added to the lower chamber. After 48 h, cells on the bottom of the inserts were fixed in 4% paraformaldehyde and stained with 0.05% crystal violet. Then cells that invaded into the lower surface were counted. Each experiment was repeated at least three times.

### RNA extraction and quantitative real-time polymerase chain reaction

Total RNA was isolated from cultured cells or tissue sections (5 to 10 of 10 μm-thick) using a Trizol standard protocol (Invitrogen, USA). For formalin-fixed, paraffin-embedded (FFPE) ccRCC samples, total RNA was extracted from 5 to 10 of 10 μm-thick tissue sections using the Ambion RecoverAll kit (Ambion, USA) according to the manufacturer’s instructions. Quantitative real-time polymerase chain reaction (qRT-PCR) was performed in triplicate in the ABI 7500 fast real-time PCR System (Applied Biosystems, USA) and normalized with U6 and Actin endogenous control. Total RNA from Normal kidney tissues was used as a control. miR-93-3p, U6 levels and endogenous mRNA levels of PEDF and Actin were detected using SYBR Green PCR Master Mix kit in accordance with the manufacturer’s instructions (Applied Biosystems, USA). U6 was used as an internal control for miR-93-3p calculation and Actin were used as an internal control for detection of PEDF mRNA. The expression of miR-93-3p and PEDF mRNA were calculated using the comparative Ct method. Relative expression intensity values were calculated as 2^-∆∆^Ct.

### Construction of PEDF 3′ untranslated region (3′-UTR) reporter plasmid

The PEDF 3′-UTR was cloned into the psi-CHECK-2 vector (Promega, USA) at 2 restriction sites for XhoI and EcoRI. Mutations were introduced by site-directed mutagenesis into putative binding sites in the 3′-UTR of PEDF gene for miR-93-3p using the TaKaRa MutanBEST Kit (Takara, Japan).

### Luciferase assays

HEK293 cells were co-transfected on 24-well plates by Lipofectamine 2000 reagent (Invitrogen, USA) with 0.5 μg of constructed reporter plasmid and miR-93-3p or control miRNA at a final concentration of 50 nM. Luciferase assays were performed using the Dual-Luciferase Reporter Assay System (Promega, USA) according to the manufacturer’s instructions.

### Protein extraction and western blot

The proteins were extracted from human ccRCC cells and specimens. Lysate was separated by 10% sodium dodecyl sulfate polyacrylamide gel electrophoresis, and the gel was blotted onto polyvinylidene fluoride (PVDF) membrane (Millipore, USA). The membrane was blocked in 5% nonfatmilk, and then incubated with either rabbit anti-human PEDF (ab180711, Abcam, USA), Bcl-2 (sc-56015, Santa, USA), Bax (sc-20067, Santa, USA), MMP9 (sc-53630, Santa, USA), MMP2 (sc-13595, Santa, USA), or Actin (3700, Cell Signaling Technology, USA). After washing, the membrane was incubated with the fluorescence-conjugated anti-mouse or anti-rabbit IgG (Invitrogen, USA). The bound secondary antibody was quantified using the Odyssey v1.2 software (LI-COR, USA) by measuring the band intensity (area×optical density) for each group and then normalized with Actin. The final results are expressed as fold changes by normalizing the data to control values.

### Immunohistochemistry

Paraffin-embedded sections of excised ccRCC specimens were immunostained for PEDF protein. Staining was performed with the streptavidin-biotin peroxidase complex method according to the manufacturer’s recommendation (Dako, Denmark). Rabbit anti-human PDEF primary antibody (Abcam, USA) was administered, followed by secondary goat anti-mouse IgG (Dako, Denmark). Negative controlswere performed throughout the entire immunohistochemistry procedure.

### Tumorigenicity assays in nude mice

5×10^6^ 786-O cells suspension was subcutaneously injected into the flank of 5-week-old female athymic BALB/c nude mice (SLAC Laboratory Animal Company, China). Then, control lentivirus or anti-miR-93-3p lentivirus delivering approximately 2×10^7^ transforming units of recombinant lentivirus were injected into mice once through the tail vein. Growth rates were determined by measuring tumor size over time. Tumor size was measured with calipers after the tumor cell injection every 7 days for a period of 4 weeks. Tumor volume was determined using the formula: volume = length × width^2^/2.

For experimental metastasis models, control and stable knockdown miR-93-3p cells (stable luciferase-transfected 786-O cells) were injected into the tail vein of nude mice. To monitor metastasis, tumors derived from stable luciferase-transfected 786-O cells were imaged to observe luciferase expression on day 28 after tumor cell injection. Briefly, the animals were anesthetized and then injected IP with luciferin at 150 mg/kg in a volume of 100 μL. Images were captured at a peak time of 15-20 min after injection using an IVIS-200 Imaging System (Xenogen Corporation, USA) and then processed using Living Image software. All animal procedures were approved by the Harbin Medical University Animal Committee.

### Statistical analysis

Statistical analysis was performed with SPSS13.0 software. Student t test, ANOVA, or chi-square analysis was applied, where appropriate. Survival rates were estimated using the Kaplan-Meier method, and survival curves were compared using the log-rank test. Survival data were evaluated by using univariate and multivariate Cox regression analyses. A probability of <0.05 (*) or <0.001 (**) was considered significant.

## Abbreviations

ccRCC: clear cell renal cell carcinoma
FFPE: formalin-fixed, paraffin-embedded
MMPs: matrix metalloproteinases
PEDF: pigment epithelium derived factor
qRT-PCR: quantitative real-time polymerase chain reaction
UTR: untranslation region
